# High baseline perivascular space volume in basal ganglia is associated with attention and executive function decline in Parkinson's disease

**DOI:** 10.1002/brb3.3607

**Published:** 2024-07-15

**Authors:** Ryan Patrick Foreman, Erin Kaye Donahue, Jared Joshua Duran, Dawn M. Schiehser, Andrew Petkus, Joseph O'Neill, Daniel Phillip Holschneider, Jeiran Choupan, John Darrell Van Horn, Ece Bayram, Irene Litvan, Michael Walter Jakowec, Giselle Maria Petzinger

**Affiliations:** ^1^ Department of Neurology, Keck School of Medicine University of Southern California Los Angeles California USA; ^2^ Alfred E. Mann School of Pharmacy and Pharmaceutical Sciences University of Southern California Los Angeles California USA; ^3^ Veterans Administration San Diego Healthcare System (VASDHS) San Diego California USA; ^4^ Department of Psychiatry University of California San Diego San Diego California USA; ^5^ Department of Psychiatry & the Behavioral Sciences University of Southern California Los Angeles California USA; ^6^ Division of Child Psychiatry UCLA Semel Institute for Neuroscience Los Angeles California USA; ^7^ Laboratory of NeuroImaging, USC Stevens Neuroimaging and Informatics Institute, Keck School of Medicine University of Southern California Los Angeles California USA; ^8^ School of Data Science University of Virginia Charlottesville Virginia USA; ^9^ Department of Psychology University of Virginia Charlottesville Virginia USA; ^10^ Parkinson and Other Movement Disorder Center, Department of Neurosciences University of California, San Diego San Diego California USA

**Keywords:** Virchow–Robinson space

## Abstract

**Background:**

Pathologic perivascular spaces (PVS), the fluid‐filled compartments surrounding brain vasculature, may underlie cognitive decline in Parkinson's disease (PD). However, whether this impacts specific cognitive domains has not been investigated.

**Objectives:**

This study examined the relationship of PVS volume at baseline with domain‐specific and global cognitive change over 2 years in PD individuals.

**Methods:**

A total of 39 individuals with PD underwent 3T T1w magnetic resonance imaging to determine PVS volume fraction (PVS volume normalized to total regional volume) within (i) centrum semiovale, (ii) prefrontal white matter (medial orbitofrontal, rostral middle frontal, and superior frontal), and (iii) basal ganglia. A neuropsychological battery included assessment of cognitive domains and global cognitive function at baseline and after 2 years.

**Results:**

Higher basal ganglia PVS at baseline was associated with greater decline in attention, executive function, and global cognition scores.

**Conclusions:**

While previous reports have associated elevated PVS volume in the basal ganglia with decline in global cognition in PD, our findings show such decline may affect the attention and executive function domains.

## Abstract 2

PVS, recently recognized as underlying PD pathophysiology, have not been explored in their contribution to specific aspects of cognitive decline. We found that perivascular spaces in the basal ganglia at baseline are predictive of cognitive decline within the executive function and attention domains in PD individuals over 2 years.

## Graphical abstract

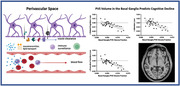

## 1 INTRODUCTION

Parkinson's disease (PD) is a progressive neurodegenerative disorder affecting both motor and nonmotor functions, including cognition (Aarsland et al., 2017). Cognitive impairment is common in PD and can affect multiple cognitive domains, including executive function, attention, memory, and visuospatial function (Aarsland et al., 2021). Cognitive impairment often progresses to dementia. Though the pathophysiology underlying cognitive changes in PD remains poorly elucidated, one potential mechanism may be dysfunction of the glymphatic system (Chen et al., 2022; Park et al., 2019). Glymphatic system dysfunction may result in decreased clearance of metabolic waste products and protein aggregates from the brain parenchyma (Debette et al., 2019; Ding et al., 2021; Zhu et al., 2010) leading to increased perivascular space volume (Xue et al., 2020; Zou et al., 2019).

The glymphatic system has several functions in healthy individuals, including (i) transport of neurotransmitters and nutrients (e.g., glucose and lipids), (ii) immune surveillance, and, as previously mentioned, (iii) clearance of cellular debris, metabolic waste products, and protein aggregates (Jessen et al., 2015). Perivascular spaces (PVS) are an essential component of this system, encompassing the fluid‐filled spaces surrounding penetrating arteries and veins in the brain. These spaces serve as the primary influx and efflux pathways for essential nutrients and waste products, alike. PVS are found in both white matter and gray matter, including in the basal ganglia. PVS enlargement, quantified on magnetic resonance imaging (MRI) as either greater PVS volume or higher incidence of dilated PVS, is thought to indicate glymphatic system dysfunction, quantified in several studies as higher PVS volume or greater incidence of dilated PVS on MRI (Chen et al., 2022; Donahue et al., 2021; Park et al., 2019; Ramirez et al., 2022).

Two previous studies have linked basal ganglia PVS to general decline of cognitive function in PD. The first (Park et al., 2019) associated higher basal ganglia PVS in PD individuals with conversion from normal cognition to mild cognitive impairment (MCI) or from MCI to dementia over 5 years. The second associated higher PVS in the basal ganglia in PD with greater decline in Montreal Cognitive Assessment (MoCA) scores over 3 years (Chen et al., 2022). While these studies support an association between PVS and decline in general cognition in PD, the relationship between PVS and decline in specific cognitive domains has yet to be examined.

The primary goal of this study was to evaluate PVS volume, expressed as volume fraction, within white matter and the basal ganglia and its association with cognitive decline within specific cognitive domains. PVS volume was determined from T1w MRI using a previously described method to obtain volume fraction measurements (Donahue et al., 2021). Along with white matter within the centrum semiovale, prefrontal white matter regions (medial orbitofrontal, rostral middle frontal, and superior frontal) were also selected for analysis due to their known role in PD‐cognition (Farina et al., 2000; Lewis et al., 2003; Taylor et al., 1990). A full neuropsychological assessment was collected on study participants at baseline and after 2 years.

## 2 METHODS

### 2.1 Study participants

A total of 39 participants with idiopathic PD and relevant PVS and lifestyle data (Table 1) were included in this cross‐sectional analysis. Participants were evaluated as part of a multi‐site (University of Southern California (*n* = 19) and Veterans Affairs San Diego Healthcare Systems/University of California at San Diego (*n* = 20)) study. PD diagnosis was based on the UK Brain Bank criteria (Hughes et al., 1992). Inclusion criteria included (i) being willing and able to provide informed consent; (ii) being medically eligible for MRI; and (iii) having Hoehn and Yahr score ≤III. Exclusion criteria included (i) history of severe psychiatric illness or other non‐PD neurological disease and (ii) dementia based on International Parkinson and Movement Disorders Society (MDS) PD‐dementia criteria (Emre et al., 2007). Institutional review boards from each site approved all study procedures. Demographic data were collected, including age, sex, body mass index (BMI; kg/m^2^), and cardiovascular risk score (summing 0 = no/1 = yes for smoking, hypertension, high cholesterol, or diabetes) at baseline. MDS‐Unified Parkinson's Disease Rating Scale (MDS‐UPDRS) part III score (motor) and levodopa equivalent dosage (LED; mg) (Parkinson's Disease Measurement, 2010) were collected at baseline and 2 years. All assessments were conducted in the “ON” medication state.

**TABLE 1:** Study demographics.

	Baseline mean (SD)	2‐Year mean (SD)
N (female)	39 (Manjón et al., 2010)	
Age	65.13 (8.84)	
Body mass index (kg/m^2^)	25.54 (4.16)	
MDS‐UPDRS motor score	20.38 (9.24)	25.64 (15.02)
Levodopa equivalent dosage (mg)	581.13 (383.53)	746.49 (547.22)
Cardiovascular risk score	0.56 (0.75)	
Site	19 USC/20 UCSD	

*Note*: Data are presented as mean (standard deviation).

Abbreviations: MDS, Movement Disorders Society; UCSD, University of California San Diego; UPDRS, Unified Parkinson's Disease Rating Scale; USC, University of Southern California.

### 2.2 Perivascular space mapping

Structural MRI was acquired on a Siemens Prisma (USC) or a GE MR 750 (UCSD) 3T MRI scanner. Whole‐brain T1w MRI was acquired with a magnetization‐prepared rapid gradient‐echo pulse sequence (flip 8°, repetition time/echo time/inversion time = 2400/2.22/1000 ms, voxels 0.8 × 0.8 × 0.8 mm^3^; 1 NEX, acceleration factor 2, 6:38 min). Study site (USC or UCSD) was included as a covariate to account for any differences due to scanner type.

T1w MRI from baseline study visits was preprocessed and regionally parcellated with FreeSurfer v5.3.0. Preprocessing included motion correction, nonuniform intensity normalization, Talairach transformation, and skull stripping. PVS was mapped from T1w MRI as previously described (Sepehrband et al., 2019). Filtering was applied to remove noise that was not spatially repeated (Manjón et al., 2010). A Frangi filter was applied to estimate a “vesselness” measure for each voxel, with parameter c set to half the value of the maximum Hessian norm (Cabeen, 2020; Frangi et al., 1998). The scale was set to a range of 0.1–5 voxels to maximize vessel inclusion. A brain‐wide quantitative map of vesselness was produced with a threshold of 0.0002 to obtain the PVS mask. All T1w MRI were manually checked for misalignment and post‐processing failure and corrected for erroneously mapped PVS (Sepehrband et al., 2021). Regional masks were derived for the basal ganglia and for the white matter subjacent to each cerebral cortical region in the Desikan–Killiany atlas using Freesurfer's recon‐all module (Desikan et al., 2006). A centrum semiovale mask was prepared by merging the subcortical white matter masks for all the regions in Table S1. All PVS masks were meticulously inspected by trained operators (J. C., E. K. D., R. P. F., and J. J. D.) to remove any white matter hyperintensities or lacunes erroneously labeled as PVS by the automated pipeline. For basal ganglia, centrum semiovale, and the individual medial orbitofrontal, rostralmiddle frontal, and superior frontal white matter regions, PVS volume within each region was normalized to the total volume of the same region. The resulting ratio was termed “volume fraction.” All PVS results below are expressed as volume fraction.

### 2.3 Neuropsychological assessment

Cognitive tests were conducted at baseline and follow‐up visits (approximately 2 years after baseline). The neuropsychological battery measured global cognitive performance and performance across cognitive domains including executive function, memory, visuospatial function, attention, and language, calculating domain scores as previously described for both baseline and follow‐up using mean and standard deviation from baseline (Donahue, Venkadesh, et al., 2022). Cognitive change scores were calculated by subtracting baseline domain scores from follow‐up domain scores, such that individuals with cognitive decline had negative change scores. All cognitive change scores were normalized to time between visits to control for differing period between study visits by dividing change scores by the days between baseline and follow‐up visit. This method assumes that the relationship between time and cognitive change is linear.

### 2.4 Statistics

All statistical analyses were conducted using SPSS, Version 28.0.1 (IBM Corp.). A bivariate unadjusted Pearson correlation was performed between the cognitive change score in each domain and global cognition and the baseline PVS volume fraction in each brain region (centrum semiovale, medial orbitofrontal, rostral middle frontal, superior frontal, and basal ganglia). If a correlation was significant, a hierarchical linear regression analysis was run using the following covariates: age, sex, change in MDS‐UPDRS part III score, BMI, change in LED, cardiovascular risk score, and study site (1 = USC, *n* = 19 and 2 = UCSD, *n* = 20). All reported regression *p*‐values are Benjamini‐Hochberg False Discovery Rate corrected within each predictor variable to account for any possible false positives resulting from multiple comparisons. Any individual beyond three standard deviations in either the independent or dependent variable was considered an extreme outlier and was excluded from analyses for that comparison. (Three subjects were removed from the medial orbitofrontal and one from the basal ganglia analyses.)

## 3 RESULTS

The total number of participants in this study was 39. The total age of the participants was on average 65.13 years at baseline (SD = 8.84), 49% were male, with an average baseline body mass index (BMI) of 25.54 kg/m^2^ (SD = 4.16). Participants had an average baseline MDS‐UPDRS motor score (part III) of 20.38 (SD = 9.24), an average follow‐up MDS‐UPDRS motor score (part III) of 25.64 (SD = 15.02), and an average change in MDS‐UPDRS motor score (part III) of −5.275 (SD = 14.29). Participants had an average baseline LED of 581.13 mg (SD = 383.53), an average follow‐up LED of 746.49 (SD = 547.22), and an average change in LED of 173.05 (SD = 379.41). Participants had an average cardiovascular risk score of 0.56 (SD = 0.75), with 19 subjects at the University of Southern California (USC) and 20 subjects at the University of California San Diego (UCSD) (Table 1). PVS volume fraction distributions for centrum semiovale, rostral middle frontal, superior frontal, medial orbitofrontal, and basal ganglia assessed are found in Figure S1.

Correlation analysis revealed that PVS volume fraction in the basal ganglia at baseline was negatively associated with change in global cognition (*r*(38) = −0.373, *p* = 0.019), attention (*r*(38) = −0.397, *p* = 0.012), and executive function (*r*(38) = −0.351, *p* = 0.029) (Table S2). No other associations were observed with baseline basal ganglia PVS and change in language, visuospatial, and memory domains (all *p*’s ≥ 0.128; Table S2). No association was observed between baseline PVS volume fraction in centrum semiovale, rostral middle frontal, superior frontal, and medial orbitofrontal regions and cognitive change in any domain (all *p*’s ≥ 0.128; Table S2). In an exploratory analysis, we tested correlations separately for right hemispheric and left hemispheric PVS volume fraction in the centrum semiovale and cognitive change in each of the five cognitive domains, as well as change in global cognition. This did not result in any significant associations.

**FIGURE 1:**
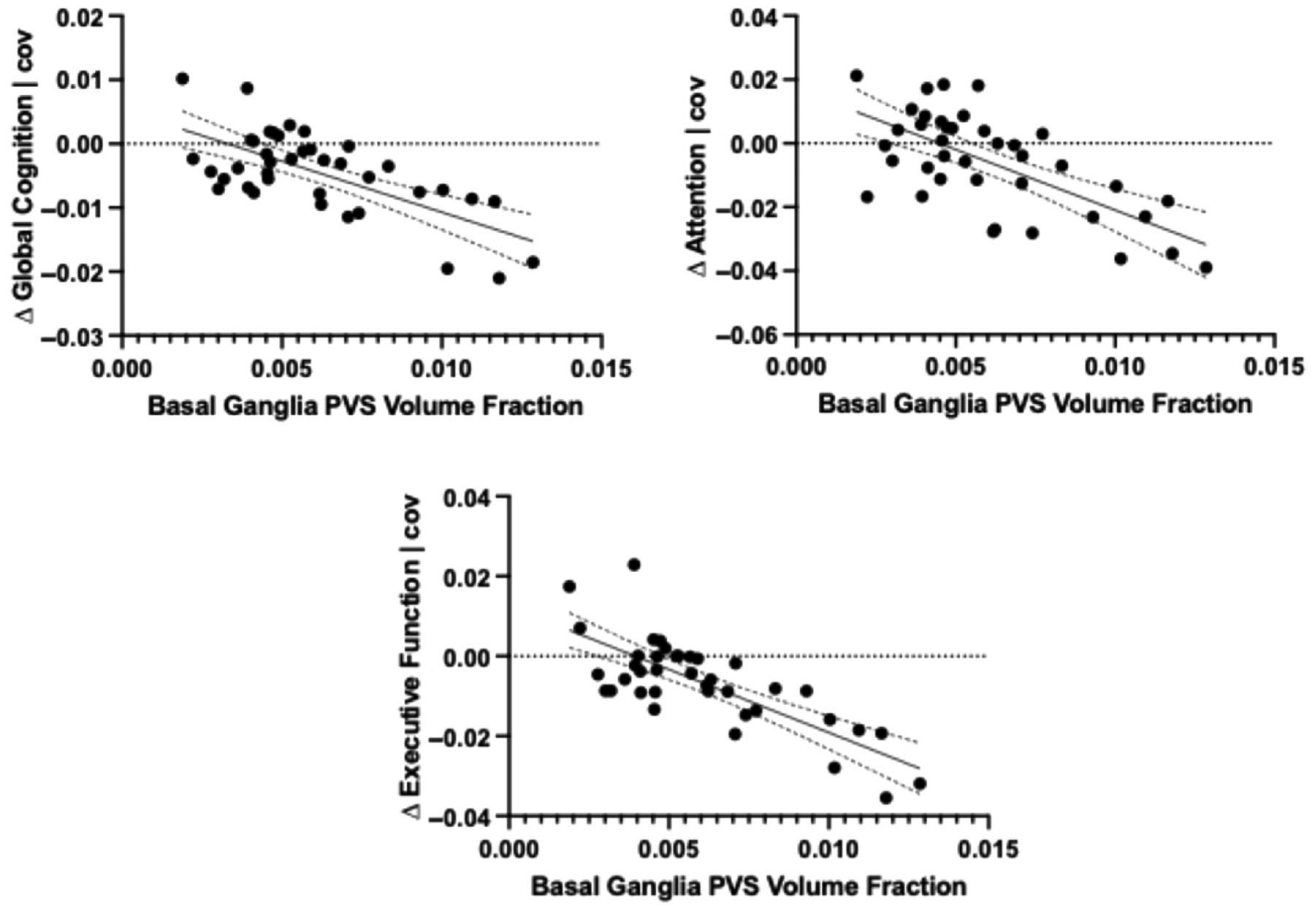
Significant adjusted association between baseline perivascular space (PVS) in the basal ganglia and cognitive change over 2 years in Parkinson's disease. Cognitive change scores are normalized to time between visits. Corrected for age, sex, change in Movement Disorders Society‐Unified Parkinson's Disease Rating Scale (MDS‐UPDRS) part III score, body mass index (BMI), change in levodopa equivalent dosage (LED), cardiovascular risk score, and study site.

After adjusting for age, sex, BMI, change in MDS‐UPDRS part III score, cardiovascular risk score, study site, and change in LED, baseline basal ganglia PVS volume fraction remained significantly associated with change in global cognition (unstandardized *β* = −1.676, 95% confidence interval [CI] [−3.229, −0.124], Δ*R* ^2^ = 0.118, *p* = 0.035; Table S3; Figure 1), change in attention (unstandardized *β* = −3.154, 95% CI [−6.277, −0.032], Δ*R* ^2^ = 0.093, *p* = 0.048; Table S3; Figure 1), and change in executive function (unstandardized *β* = −3.415, 95% CI [−6.729, −0.101], Δ*R* ^2^ = 0.119, *p* = 0.044; Table S3; Figure 1). In no region did PVS volume fraction correlate with the change in MDS‐UPDRS motor score or with the change in LED.

## 4 DISCUSSION

The aim of this study was to investigate whether higher PVS volume at baseline predicts cognitive decline 2 years later within specific domains in PD. Of particular interest were executive function and attention, due to their early and progressive involvement in PD. We examined PVS volume in the basal ganglia and multiple white matter regions to determine which may be more predictive of PD‐related cognitive change. We found that higher PVS volume in the basal ganglia was associated with a greater decline in attention and executive function, as well as global cognition, over a 2‐year period.

These findings accord with prior studies that demonstrated that stronger presence of basal ganglia PVS predicts greater decline in measures of general cognition in PD, including the MoCA score. While Park et al. (2019) showed that higher baseline basal ganglia PVS count in PD individuals predicted conversion from normal cognition to MCI or from MCI to dementia, Chen et al. (2022) found that higher basal ganglia PVS count in PD correlated with greater decline in MoCA score over 3 years. Our study extends these findings by demonstrating that higher PVS burden may be accompanied by cognitive decline in domains that are heavily impacted in PD, specifically executive function and attention. These domains are commonly affected early in PD and manifest progressive decline throughout the disease (Dirnberger & Jahanshahi, 2013). Basal ganglia function has been previously associated with executive function and attention, including through changes in dopamine neurotransmission (Monchi et al., 2006). Further, it has been suggested that PVS dilation is associated with the loss of dopamine neurons, though the direction of this association has not been established (Li et al., 2020). Thus, PVS dilation in the basal ganglia region may contribute to or result from pathophysiological changes in the basal ganglia, including through inflammatory processes or impaired clearance.

Unlike the basal ganglia, PVS volume in the white matter did not predict cognitive decline over 2 years in our PD sample. Prior studies have variously reported the presence or absence of a relationship between white matter PVS and cognition in PD (Chen et al., 2022; Donahue et al., 2021; Park et al., 2019). Associations between white matter PVS and cognitive decline, however, have been seen in healthy aging and in non‐PD dementia (MacLullich et al., 2004; Paradise et al., 2021). This suggests several things, including that PVS pathophysiology may vary by diagnosis. White matter PVS is thought to be more associated with proteinopathy and protein aggregate burden, while basal ganglia is more related to breakdown in structural integrity, including of the blood‐brain barrier (Li et al., 2019; Pollock et al., 1997; Roher et al., 2003). In concert, the findings to date suggest that the involvement of white matter PVS in PD‐related cognitive decline requires further investigation.

Though the mechanisms underlying PVS dilation and glymphatic system dysfunction are not well known, some studies have attempted to clarify this connection. PVS volume increase may reflect a decreased or congested flow in the glymphatic system, as demonstrated by a study that blocked glymphatic flow in rodents and saw subsequent accumulation of α‐synuclein, a pathophysiological protein hallmark of PD (Zou et al., 2019). Another proposed mechanism is neuroinflammation, which is common in PD and other neurodegenerative disorders (De Virgilio et al., 2016). A prior study of ours has identified a relationship between greater PVS burden and higher levels of putative neurometabolite markers of neuroinflammation (Donahue et al., 2022). Further studies are needed to fully elucidate the underlying mechanisms leading to increased PVS in disorders of the brain, including PD, though a relationship between PVS and disease progression, particularly cognitive decline, has become clearer.

### 4.1 Limitations and Strengths

There are several limitations to this study. The absence of T2w and FLAIR MRI volumes precluded the discernment of PVS tissue from white matter hyperintensities and lacunes by means of the automated mapping pipeline. Instead, all PVS masks were quality control checked by trained operators (J. C., E. K. D., R. P. F., and J. J. D.) to remove any white matter hyperintensities or lacunes erroneously mapped as PVS. We evaluated only patients with early PD. The association between PVS and cognition may differ in more advanced stages of the disorder. The relatively low number of participants in this study coupled with the low average PVS volume fraction observed in the white matter regions may have limited our power to detect significance in these regions. Future studies will consider a larger sample size to address these limitations. As metric of PVS burden, we used automated PVS volume fraction rather than manual PVS count. Prior work, however, has found good agreement between automated and manual quantification of PVS (Sepehrband et al., 2019), while the latter is intrinsically more subjective. Note further that the PVS count does not reflect the dilation of individual lesions, which may be an additional indicator of glymphatic dysfunction. This study did not include a healthy control group. Future studies will compare these findings with healthy controls. This study normalized change scores by the number of days between visits to help control for the variance in follow‐up intervals. This approach assumes that the relationship between time and cognitive decline is linear, which may not be accurate in PD. We feel our study population is representative of the general PD patient population, with patients being recruited from two different movement disorders clinics and diagnosed by experts. However, to improve generalizability in studies of this nature we must work to improve harmonization of PVS analysis throughout the field. Strengths of this study include the utilization of automated (objective) assessment of PVS and evaluation of individual cognitive domains, providing more specific evidence of how PVS impacts cognitive decline.

## 5 CONCLUSIONS

This study provides evidence that greater PVS volume in the basal ganglia in early PD is associated with increased cognitive decline in executive function, attention, and global cognition in PD. PVS thus represents a possible therapeutic target to forestall PD‐related cognitive decline. Future studies should investigate the role of PVS in advanced PD including individuals with PD‐dementia.

## AUTHOR CONTRIBUTIONS

**Ryan Patrick Foreman**: Data curation; formal analysis; investigation; methodology; resources; visualization; writing—original draft; writing—review and editing. **Erin Kaye Donahue**: Conceptualization; data curation; formal analysis; investigation; methodology; resources; visualization; Writing—original draft; Writing—review and editing. **Jared Joshua Duran**: Data curation; resources. **Dawn M. Schiehser**: Data curation; formal analysis; investigation; methodology; resources; supervision; validation; writing—review and editing. **Andrew Petkus**: Data curation; formal analysis; investigation; methodology; resources; writing—review and editing. **Joseph O'Neill**: Conceptualization; data curation; formal analysis; investigation; methodology; resources; software; supervision; validation; visualization; writing—review and editing. **Daniel Phillip Holschneider**: Data curation; investigation; resources; writing; review and editing. **jeiran choupan**: Conceptualization; data curation; investigation; methodology; resources; software. **John Darrell Van Horn**: Conceptualization; data curation; formal analysis; investigation; methodology; resources; software; supervision; validation; visualization; writing—review and editing. **Ece Bayram**: Data curation; investigation; resources; writing—review and editing. **Irene Litvan**: Data curation; resources; writing—review and editing. **Michael Walter Jakowec**: Conceptualization; data curation; formal analysis; investigation; methodology; project administration; resources; supervision; validation; visualization; Writing—original draft; writing—review and editing. **Giselle Maria Petzinger**: Conceptualization; data curation; formal analysis; funding acquisition; investigation; methodology; project administration; resources; supervision; validation; visualization; Writing—original draft; writing—review and editing

## CONFLICT OF INTEREST STATEMENT

The authors declare no conflicts of interest.

### PEER REVIEW

The peer review history for this article is available at https://publons.com/publon/10.1002/brb3.3607

## Supporting information


**Supplementary Table 1**: White matter regions listed in the Desikan‐Killiany atlas included in centrum semiovale PVS calculation.
**Supplementary Table 2**: Pearson correlations between baseline regional volume fractions of perivascular space and cognitive change scores from baseline to two years in Parkinson's disease individuals. Cognitive change scores are normalized to time between visits.
**Supplementary Table 3**: Adjusted associations between perivascular space volume fraction in basal ganglia and cognitive change over two years in Parkinson's disease. Cognitive change scores are normalized to time between visits. Corrected for age, sex, change in MDS‐UPDRS part III score, BMI, change in LED, cardiovascular risk score, and study site.
**Supplementary Figure 1**: Violin plots depicting the distribution of PVS volume fraction in each region assessed.

## Data Availability

Data are available upon reasonable request to the corresponding author.
