# Supplementary material for: High baseline perivascular space volume in basal ganglia is associated with attention and executive function decline in Parkinson's disease
Source: Brain Behav. 2024 Jul 15;14(7):e3607. doi: 10.1002/brb3.3607 (PMC11250171; doi:10.1002/brb3.3607)
Supplement: Supplementary file 1 — Supplementary Table 1: White matter regions listed in the Desikan‐Killiany atlas included in centrum semiovale PVS calculation. Supplementary Table 2: Pearson correlations between baseline regional volume fractions of perivascular space and cognitive change scores from baseline to two years in Parkinson's disease individuals. Cognitive change scores are normalized to time between visits. Supplementary Table 3: Adjusted associations between perivascular space volume fraction in basal ganglia and cognitive change over two years in Parkinson's disease. Cognitive change scores are normalized to time between visits. Corrected for age, sex, change in MDS‐UPDRS part III score, BMI, change in LED, cardiovascular risk score, and study site. Supplementary Figure 1: Violin plots depicting the distribution of PVS volume fraction in each region assessed. [file BRB3-14-e3607-s001.docx]

**Supplementary Tables**

**Supplementary Table 1**

| White matter regions included in Centrum Semiovale Calculation |
| --- |
| caudal middle frontal  inferior parietal  pars opercularis  pars orbitalis  pars triangularis  post central  precentral  rostral middle frontal  superior frontal  superior parietal  supramarginal  unsegmented white matter not defined |

All derived from Desikan-Killiany atlas

**Supplementary Table 2**

|  | Δ Global  Cognition | | Δ Language | | Δ Attention | | Δ Visuospatial  Function | | Δ Memory | | Δ Executive  Function | |
| --- | --- | --- | --- | --- | --- | --- | --- | --- | --- | --- | --- | --- |
| PVS Volume  Fraction | **r** | ***p*** | **r** | ***p*** | **r** | ***p*** | **r** | ***p*** | **r** | ***p*** | **r** | ***p*** |
| centrum semiovale | -.090 | .581 | -.188 | .246 | -.156 | .336 | .016 | .922 | .032 | .844 | .107 | .510 |
| basal ganglia | **-.373*** | **.019** | -.248 | .128 | **-.397*** | **.012** | .197 | .230 | -.112 | .496 | **-.351*** | **.029** |
| medial orbitofrontal | .029 | .864 | -.097 | .562 | -.150 | .368 | .034 | .841 | .195 | .240 | .145 | .386 |
| rostral middle frontal | -.090 | .581 | -.188 | .246 | -.156 | .336 | .016 | .922 | .032 | .844 | .107 | .510 |
| superior frontal | -.098 | .548 | -.163 | .316 | -.155 | .341 | -.004 | .982 | -.020 | .904 | .120 | .461 |

*PVS: perivascular space*

**Supplementary Table 3**

|  |
| --- |

|  |  | **95% CI** |  |
| --- | --- | --- | --- |
|  | **β** | **Lower, Upper** | ***p*** |
| Basal Ganglia PVS Volume Fraction | | | |
| ∆ Global Cognition | -1.676 | -3.229, -.0124 | .035 |
| ∆ Attention | -3.154 | -6.277, -0.032 | .048 |
| ∆ Executive Function | -3.415 | -6.729, -0.101 | 0.44 |

Supplementary Figure 1
